# Antimicrobial and Antibiofilm Properties of Bioceramic Materials in Endodontics

**DOI:** 10.3390/ma14247594

**Published:** 2021-12-10

**Authors:** Zhejun Wang, Ya Shen, Markus Haapasalo

**Affiliations:** Division of Endodontics, Department of Oral Biological and Medical Sciences, Faculty of Dentistry, The University of British Columbia, Vancouver, BC V6T 1Z3, Canada; zhejun@dentistry.ubc.ca (Z.W.); yashen@dentistry.ubc.ca (Y.S.)

**Keywords:** bioceramic material, biofilm, root repair material, sealer

## Abstract

Microbes are prevalent in the root canals of necrotic teeth, and they are the cause of primary and post-treatment apical periodontitis. Bacteria can dwell within the infected root canal system as surface-adherent biofilm structures, which exhibit high resistance to antimicrobial agents. Bioceramic materials, with their biocompatible nature and excellent physico-chemical properties, have been widely used in dental applications, including endodontics. This review focuses on the application of bioceramic technology in endodontic disinfection and the antibiofilm effects of endodontic bioceramic materials. Different bioceramic materials have shown different levels of antibiofilm effects. New supplements have emerged to potentially enhance the antibiofilm properties of bioceramics aiming to achieve the goal of microbial elimination in the root canal system.

## 1. Introduction

Microorganisms and their by-products are the main etiological factors responsible for pulpal and periapical diseases [1]. Microorganisms found in the root canals are known to form biofilms, which renders them more resistant to antimicrobial agents than bacteria in the planktonic state [2]. The purpose of endodontic treatment is to prevent microbial contamination of the root canal system and to eliminate the microbes from the infected root canal in order to achieve clinical and radiographic success [3]. Although chemo-mechanical preparation significantly reduces microorganisms inside the infected root canal system, proportionally large areas of the main root canal wall remain untouched by the instruments, and it is virtually impossible to completely remove 100% of the microbes by irrigation and other methods [4]. Different methods of irrigant agitation have been introduced, including pipetting, heating the irrigant [5], sonic and ultrasonic activation [6], and multisonic activation [7]. These methods have been effective in promoting the movement of the irrigant to clean areas that are beyond the reach of the instrumentation. Beyond the irrigant agitation, the use of endodontic materials such as sealers, cements, pastes, putty, and obturation materials with antibiofilm activity is considered beneficial in further reducing the residual microorganisms and preventing leakage of a potential reinfection [8].

Bioceramics are inorganic, non-metallic, biocompatible materials that are used in direct contact with living tissues in the medical and dental fields [9]. Since they are chemically stable, non-corrosive, and interact well with organic tissues, more bioceramic materials have been developed and successfully used in endodontic treatments, including pulp capping, obturation, apical barrier formation, perforation repairs, and root-end filling [10]. Some endodontic bioceramics are powder/liquid systems requiring manual mixing, and some are premixed materials requiring moisture from surrounding tissues to set. The setting process allows the bioceramics to achieve a superior seal with the tooth structure [11].

The antimicrobial and antibiofilm properties are exerted during the setting process by increasing the pH and ion release from the material [12]. A high-quality seal and antimicrobial properties are both critical to achieving success in endodontic treatment [13]. Antibiofilm properties may continue to exist in a bioceramic-treated environment via further physico-chemical reactions (e.g., biomineralization effect) with the surrounding dental hard tissues [14]. Meanwhile, challenges remain in balancing the antibiofilm properties with biocompatibility in the development of current bioceramics for endodontics. This review takes a critical look at the antimicrobial and antibiofilm properties of currently available bioceramic materials used in endodontics and looks forward to embracing possible new technologies that may facilitate the elimination of all microorganisms for long-term endodontic success.

## 2. Endodontic Biofilm Formation

The formation of oral biofilms has a dynamic nature as a linear process that commences when free-floating bacterial cells attach to the tooth surface [15]. This attachment is followed by embedding in a self-produced matrix of extracellular polymeric substances and exhibits an altered phenotype with respect to growth rate and gene transcription of the biofilm bacteria compared with their planktonic counterparts [16]. These various phases of microbial interactions with the surface require the production of extracellular polymers that assist in initial adhesion, maintenance of the biofilm three-dimensional structure, and detachment from matrix-enclosed aggregates [17]. Microorganisms in biofilms are more resistant to elimination than those in planktonic form [18,19].

**Figure 1:**
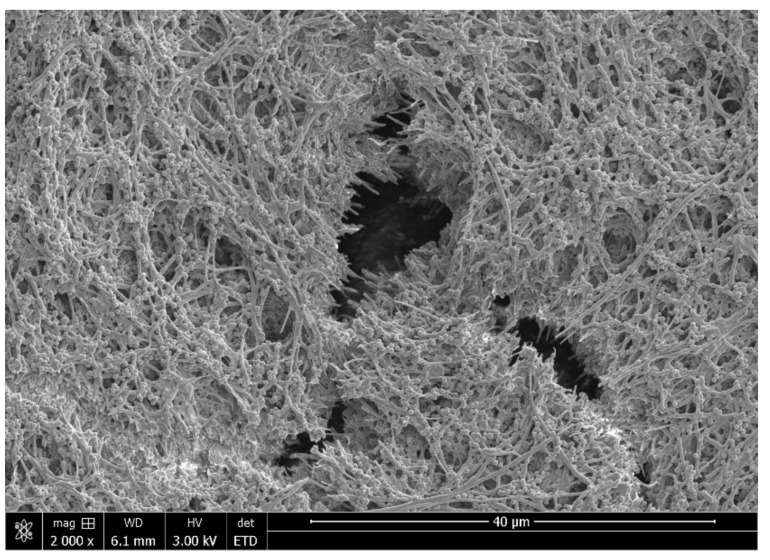
Scanning electron microscope image of multi-species mature plaque biofilm grown on collagen-coated hydroxyapatite disk.

**Figure 2:**
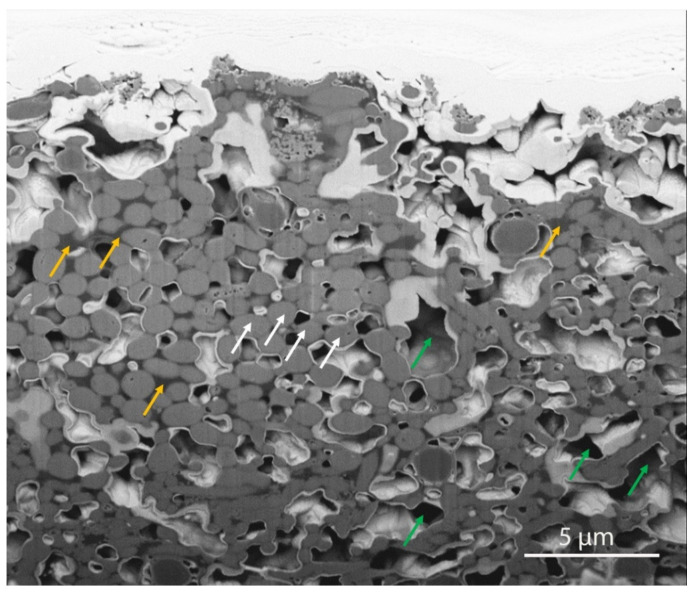
FIB-SEM image of a milled mature plaque biofilm composed of bacterial cells (white arrows), extracellular polymeric substances (yellow arrows) and micro water channels (green arrows).

Most endodontic infections originate from plaque on the tooth surface and gingival crevice/periodontal pocket [20]. In the anaerobic microenvironment of endodontically infected teeth, the microorganisms colonize root canal space, bind to the root canal wall, and grow as habitat-adapted multi-species endodontic biofilms (Figure 1) [16]. An extraradicular biofilm formation could also be observed from the external root surface [21]. Regular scanning electron microscopy (SEM, Helios Nanolab 650, FEI, Eindhoven, The Netherlands) shows bacterial aggregates on the biofilm surface. A focused ion beam scanning electron microscope (FIB-SEM, Helios Nanolab 650, FEI, Eindhoven, The Netherlands) has been introduced as a useful micromachining tool for biological samples [22]. The FIB-SEM is capable of milling to remove sections for viewing of features below the readily observable surface layer [23]. Dental plaque biofilm cell layers and the abundance of extracellular polymeric substance matrix associated with bacterial cells are shown under the FIB-SEM after sequential milling (Figure 2). It is known that certain bacteria can attach to type I collagen in dentin through the expression of surface adhesins [24]. Dentinal tubules are the tunnels for bacterial invasion from the main root canal towards the dentin periphery to induce deep and persistent dentin infection [25]. Bacteria hiding in the dentin tubules are difficult to reach and eliminate by traditional disinfecting strategies, such as instrumentation and irrigation.

Bioceramic materials have been used in a variety of endodontic applications, including vital pulp therapy, sealer-based obturation, root-end fillings, regenerative endodontics, etc. Regardless of whether used alone or together with other materials, bioceramics play an important role in adapting to the surrounding tissues and materials [14]. Innovations have been continually developed in bioceramic materials with the goal of not only being biocompatible, but also acting as a synergistic antimicrobial component against established bioceramics and the potential future formation of new endodontic biofilms [10].

## 3. Bioceramic Root Repair Material

### 3.1. MTA

**Figure 3:**
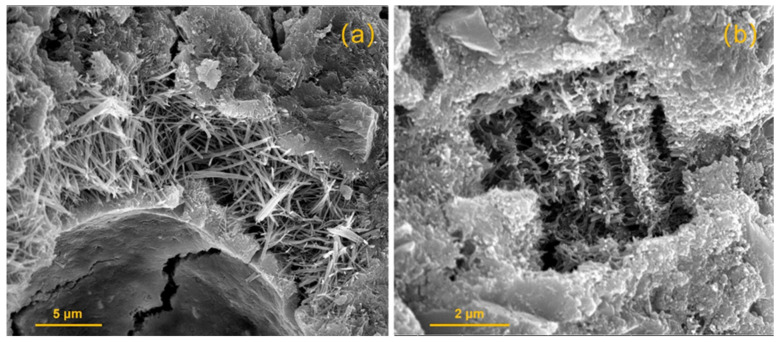
SEM image of cross-section of (**a**) MTA and (**b**) EndoSequence root repair material putty exposed to butyric acid at pH 7.4 after 7 days of setting.

Mineral trioxide aggregate (MTA) (Dentsply-Tulsa Dental, Johnson City, TN, USA) is one of the most commonly used biomaterials in endodontics based on its excellent biological, chemical, and physiological properties (Figure 3a). Mineral trioxide aggregate contains a mixture of dicalcium silicate, tricalcium silicate, tricalcium aluminate, gypsum, tetracalcium aluminoferrite, and bismuth oxide [11]. MTA has been widely used for vital pulp therapy, apical barrier formation, repair of root perforations, and root-end filling. The antibiofilm properties of MTA have been extensively evaluated using different strains of microbes/biofilms that present in the oral cavity and using different microbiological testing methods [11].

The reason why a tricalcium silicate-based cement, such as MTA, has antibiofilm activity is mostly due to the pH increase during the setting process. The pH can increase from the formation of calcium hydroxide during the hydration reaction of MTA. Torabinejad et al. [26] showed that the pH of MTA is 10.5 at the time of mixing and can increase up to 12.9 after 3 h of setting. High pH affects the structure of the endodontic bacterial cells by causing DNA degradation and cellular protein damage, resulting in the decrease of cellular viability [27,28].

*Enterococcus faecalis* biofilm has been claimed to be unable to survive if the pH values were set to higher than 11.5 [29]. MTA also exhibited antimicrobial activity on other types of bacterial biofilms, including *Staphylococcus aureus*, *Escherichia coli, Pseudomonas aeruginosa*, and *Porphyromonas gingivalis* [30]. As well, a recent study showed ProRoot MTA (Dentsply-Tulsa Dental, Johnson City, TX, USA) had antimicrobial activity against *Candida albicans* [31]. Similar antifungal effects were reported in previous studies [32,33]. Dissimilar results from different studies on the antibiofilm activities of MTA may be attributed to the composition of the biofilms and the amount of material applied [11].

Recently, a “tooth-on-a-chip” model was developed to enable direct visualization and analyses of bioceramic cement interactions with oral biofilms [34]. ProRoot MTA was applied using *S. mutans* biofilm to simulate microbial infection at the biomaterial-dentin interface. ProRoot MTA disrupted the integrity of the biofilm and over 90% of bacterial cells within the biofilms were killed both at the biofilm’s outer and inner layers [35]. The antibiofilm effect was likely due to the high pH microenvironment and calcium ions released from ProRoot MTA [35].

In clinical reality, MTA commonly interacts with an environment of blood, tissue fluid, and dentin structure. A recent study showed ProRoot MTA killed more bacteria when it was in contact with water than when it was in contact with heparinized human blood [36]. Moreover, the antibiofilm activity of MTA was significantly reduced after aging for 7 days in water and blood using a direct contact test and an intra-tubular infection test [36].

### 3.2. Other MTA-Based Cements

Numerous MTA-based cements have been developed, aiming to retain the excellent physico-chemical and biological properties of MTA and overcome the limitations of traditional MTA, e.g., long setting time, handling difficulty, and staining.

One example is NeoMTA Plus (NuSmile, Houston, TX, USA), which is a calcium silicate cement developed based on the MTA formulation, containing tantalum oxide as a radiopaque element. MTA Angelus (Angelus, Londrina, PR, Brazil) [37] and MTA Repair HP (Angelus, Londrina, PR, Brazil) [38] are MTA-based cements from Brazil. MTA Angelus was reported to have similar antimicrobial activity against *E. faecalis*, *S. mutans*, *Micrococcus luteus*, *S. aureus*, *P. aeruginosa*, and *C. albicans* compared to regular MTA [11]. MTA Repair HP was reported to have a short setting time and antimicrobial activity against planktonic *E. faecalis* [39]. A new bioceramic cement White-MTAFlow (Ultradent Products Inc., South Jordan, UT, USA) showed similar antimicrobial properties to ProRoot MTA [30].

Jardine et al. [14] showed that MTA Angelus and NeoMTA Plus were not effective against multi-species biofilm using an intraoral-infected dentin biofilm model. A more recent study also showed MTA Angelus and NeoMTA Plus did not disrupt the multilayer structure formed by dual-species biofilm (*E. faecalis* and *C. albicans*) [19]. The possible reasons for their weak antibiofilm activity could be due to the methods used in these studies. The endodontic cements used in this study were set before applying on the biofilm. Heyder et al. [40] indicated that the antibacterial behavior of MTA could only be detected when freshly mixed using a direct contact test. Therefore, it is possible that the antibiofilm activity of MTA-based materials could be diminished after setting.

In addition, dentin block models were used in these recent studies, which mimics the clinical reality by direct contact of the material with multi-species biofilm in human dentin [25]. Multi-species biofilms grown in clinical-simulated conditions were more difficult to kill than planktonic bacteria [41]. Moreover, the use of confocal laser scanning microscopy and viability staining allows a three-dimensional evaluation of the viability of the biofilm adhered to dentin [14,25].

### 3.3. Biodentine

The new generation of tricalcium silicate cements contains modifications aimed to eliminate the limitations of MTA and enhance its physico-chemical properties [42]. Biodentine (Septodont, Saint-Maur-des-Fosses, France) is a fast-setting calcium silicate-based material and is recommended for use as a dentin substitute that can be used as a coronal restoration material, perforation repair material, or pulp-capping material [43,44]. Biodentine contains tricalcium silicate, calcium carbonate, zirconium oxide, and calcium chloride [10]. Zirconium oxide has been used instead of bismuth oxide as a non-inducing discoloration radiopacifier in Biodentine [45].

The antimicrobial activity of Biodentine has been shown to be dependent on calcium ion release [46,47]. The setting process of Biodentine also allowed the increase of pH values [30,48]. The alkaline and calcium release properties allowed Biodentine to kill *E. faecalis* in a direct contact test [39]. Another recent study showed Biodentine had excellent antimicrobial activity upon initial contact with the bacteria [49]. However, confocal laser scanning microscopy results have shown Biodentine to lose its antimicrobial activity after prolonged exposure to biofilms [49]. The reduction in pH value over time may contribute to the loss of antibiofilm properties as time progresses [49]. Biodentine has also been reported to be ineffective against multi-species biofilm [14]. Incorporating titanium tetrafluoride to Bioodentine has been reported to enhance its antimicrobial properties [50].

### 3.4. EndoSequence Root Repair Material

EndoSequence root repair material (Brasseler USA, Savannah, GA, USA) is a premixed putty or syringeable paste mainly composed of calcium silicate, calcium phosphate, zirconium oxide and tantalum oxide [10]. As an alternative to MTA, EndoSequence root repair material has been considered bioactive, which is the ability of a material to form an apatite-like precipitate on its surface when brought into contact with tissue fluids [51].

Antunes et al. [52] showed that MTA and EndoSequence root repair material putty had similar sealing ability. Both materials had detectable viable bacteria in the root canals in a bacterial (*E. faecalis*) nutrient leakage model after a 30-day incubation period [53]. EndoSequence root repair material demonstrates its antibacterial activity during its setting reaction due to its highly alkaline pH and mineral ion release [5]. EndoSequence root repair material putty (Figure 3b) was used as a retro-fill material in a clinical study using photodynamic therapy to disinfect the cutting surface and root-end cavity in apicoectomy surgeries. The 12–21-month success rate was reported to be up to 93% [49]. Liu et al. showed there was no significant difference in terms of antimicrobial activity against *E. faecalis* between iRoot BP Plus and MTA [54].

EndoSequence root repair material has been available in two different consistencies, including a fast-set putty and a regular-set paste. A direct contact test showed that EndoSequence root repair material putty and paste possessed antimicrobial properties against 10 clinical strains of *E. faecalis* during their setting time [55]. Moreover, EndoSequence root repair material and MTA had comparable antifungal activity against *C. albicans* biofilm [56].

## 4. Bioceramic Endodontic Sealers

### 4.1. Premixed Bioceramic Sealers

Three premixed calcium silicate-based sealers with similar chemical composition have been developed, including iRoot SP (Innovative Bioceramics, Vancouver, BC, Canada), EndoSequence BC sealer (Brasseler USA, Savannah, GA, USA) and EndoSequence BC sealer HiFlow (Brasseler USA, Savannah, GA, USA) [57,58]. Calcium silicates, calcium phosphate monobasic, zirconium oxide, tricalcium silicate, dicalcium silicate, and calcium hydroxide are the main components [10]. These three premixed sealers have shown biocompatibility, excellent sealing ability, and antimicrobial activity [59]. Bioceramic sealers have the advantage of prolonged antimicrobial activity [60]. Other types of sealers (e.g., epoxy resin-based sealers) can lose antimicrobial activity after setting [61]. A bioceramic sealer was reported to have long-lasting antimicrobial ability for up to 30 days due to the biomineralization process induced by calcium silicates/phosphates from the sealer and from the dentin minerals [62]. Moisture from dentin promotes the hydration reaction to produce calcium silicate hydrogel and calcium hydroxide to elevate and maintain a high pH in the root canal environment [60]. Silica dissolved in a high pH environment may directly inhibit bacterial viability [63].

Fresh iRoot SP was reported to have the highest pH value in the experimental periods among seven different sealers, and iRoot SP efficiently killed *E. faecalis* using a direct contact test [60]. Confocal laser scanning microscopy also showed effective killing of *E. faecalis* in dentinal tubules by freshly mixed EndoSequence BC sealer [64]. Another recent study showed that EndoSequence BC sealer exhibited significant antimicrobial capacity against an 8-week-old *E. faecalis* biofilm in a dentin infection model [65]. In contrast, a previous study reported no antimicrobial effect from EndoSequence BC sealer to *E. faecalis* under scanning electron microscopy [66]. The controversy from different studies may be due to the different methodologies applied. Scanning electron microscopy is a qualitative approach, which has limited field of view for the visualization of the overall biofilm growth on the substrate in high magnification [66]. The evaluation of the killing effect may then be limited to a small area when using SEM [66]. In contrast, confocal laser scanning microscopy with viability staining in the previous study allowed multiple areas of scanning on each sample in low magnification [64]. A more general assessment of the *E. faecalis* biofilm killing effect could be applied [64]. Quantitative results could also be obtained from confocal data using three-dimensional reconstruction software. EndoSequence BC sealer was shown to have similar antibiofilm effect against *E. faecalis* in a dentin infection model compared to AH-Plus sealer [62]. However, AH-Plus had higher antifungal activity than iRoot SP against *C. albicans*.

Another premixed calcium silicate sealer, TotalFill BC (FKG Swiss Endo, La Chaux-de-Fonds, Switzerland), killed over 40% of *E. faecalis* biofilm in dentin [67], and showed effective antimicrobial properties against single-species [68] and multi-species endodontic biofilm using a direct contact test [69,70] and confocal laser scanning microscopy [71]. One recent study showed TotalFill BC had higher antimicrobial efficacy than AH-Plus sealer using a modified direct contact test [70].

### 4.2. BioRoot RCS

BioRoot RCS (Septodont, Saint-Maur-des-Fosses, France) is a powder/liquid hydraulic cements composed of tricalcium silicate, zirconium dioxide, and povidone in the powder. The liquid contains aqueous solution of calcium chloride and polycarboxyl [72]. Possibly due to calcium release and biomineralization properties, BioRoot RCS had average short-term but superior long-term antimicrobial and antibiofilm effects [72]. A recent confocal laser scanning microscopy study showed BioRoot RCS had increased microbial killing efficacy against multi-species biofilm compared to TotalFill BC sealer (FKG Dentaire SA, Switzerland) [71]. This was probably because BioRoot RCS was able to sustain a high-alkalinity environment for a longer time. BioRoot RCS was reported to kill both young and old *E. faecalis* biofilms [47,67]. In studies using planktonic bacteria, BioRoot RCS exhibited killing effects comparable to MTA Fillapex and Pulp Canal Sealer EWT (Kerr, Sybron Dental Specialties, MI, USA) after 6 min of contact [69]. Its planktonic killing effect was lower than TotalFill and AH-Plus (Dentsply-Tulsa Dental, Johnson City, TN, USA) after 7 days of contact [67]. However, after a 30-day exposure, BioRoot RCS killed a higher percentage of bacteria than AH-Plus or TotalFill [67].

### 4.3. MTA Fillapex

MTA Fillapex (Angelus Solucoes Odontologicas, Londrina, PR, Brazil) is a calcium silicate-based sealer with a high pH and calcium ion release ability [5]. The composition of MTA Fillapex after mixing includes mineral trioxide aggregate, salicylate resin, natural resin, bismuth, silica, and lead. The ion release of lead from fresh samples of MTA Fillapex has been reported to induce cytotoxicity to mammalian cells [10]. However, the cytotoxicity can be significantly reduced after the material is set [10]. The most previous MTA Fillapex studies have used *E. faecalis* to analyze its antimicrobial activity [72]. Due to the different methodologies, different microbial strains and experimental settings, the conclusions about the antimicrobial activity of MTA Fillapex have been heterogeneous. Some studies have shown that MTA Fillapex had lower antimicrobial efficacy than BioRoot RCS or AH-Plus against planktonic *E. faecalis* [47,69,73]. Other studies showed MTA Fillapex exhibited higher antimicrobial and antibiofilm activities in comparison with other sealers [64,74]. Similar to other endodontic sealers, MTA Fillapex may lose some of its antimicrobial activity over time. Morgental et al. [75] showed that freshly mixed MTA Fillapex did not maintain antimicrobial activity after setting against *E. faecalis* using a direct contact test. Cytotoxicity has been reported from previous biocompatibility studies on MTA Fillapex [76]. The presence of salicylate resin in MTA Fillapex could explain its cytotoxicity and the salicylate resin may also play a role in killing *E. faecalis*, which can survive in alkaline environments [77,78].

## 5. Potential Supplements to Enhance Antimicrobial Properties of Bioceramics

### 5.1. Peptides

**Figure 4:**
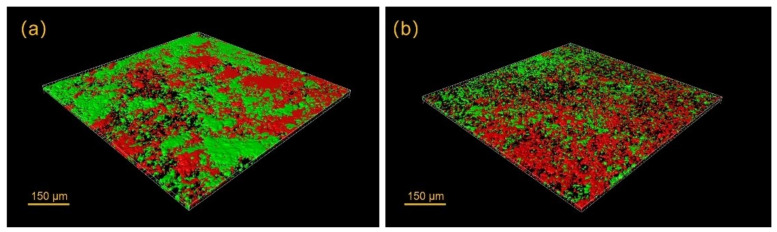
Confocal image of viability-stained plaque biofilm treated with (**a**) peptide 1018 and (**b**) peptide DJK-5. Red areas indicate killed bacteria, and green areas indicate live bacteria.

Antimicrobial peptides have recently received considerable attention for possible use in a number of dental therapeutic applications against oral biofilms [79]. Many studies have identified antimicrobial peptides as the potential next-generation alternative to traditional antimicrobial therapy in the oral cavity [80]. Peptide 1018 and DJK-5 are examples of antimicrobial peptides effective against oral biofilms (Figure 4) [81,82]. These two peptides were reported to have strong performance in inhibiting biofilm development and eradicating pre-formed oral biofilms [82,83] not only on an open biofilm platform [84], but also for single- and multi-species biofilms grown in dentinal tubules [80]. The mechanism of the antimicrobial effect by Peptide 1018 and DJK-5 peptides was by binding to and stimulating degradation the second messenger nucleotide (p)ppGpp that is involved in biofilm formation and maintenance [81,82]. Both peptides were able to bind onto the hydroxyapatite surfaces [83]. In addition, DJK-5 was also protease-resistant with enhanced antibiofilm activity [83]. Regular endodontic solutions (e.g., sodium hypochlorite, chlorhexidine, and EDTA) were used in combination with these peptides without reported side effects [85,86]. However, peptides are difficult and expensive to manufacture in large quantities due to the complex processes of their extraction, isolation, and purification [87]. The cost of using peptides as disinfectants would be impractical in the everyday clinical practice. A modified antimicrobial peptide 1018 has been reported to be able to bind to hydroxyapatite with high affinity [88]. Such types of peptides exhibited high antimicrobial and antibiofilm activity against plaque biofilm with significant biofilm volume reduction [88]. Bioceramic materials are calcium–silicate and calcium–phosphate based materials, which are biocompatible and with similar properties to dental minerals. Given the previous evidence of successful application of antimicrobial peptides on biofilms grown on hydroxyapatite and dentin, peptides have great potential to be used as supplements to be incorporated in endodontic bioceramic materials to enhance their antibiofilm properties.

### 5.2. Nanoparticles

The addition of nanoparticles to endodontic materials [89] has been reported to improve their direct and diffusible antimicrobial properties and antimicrobial activity in dentinal tubules [90]. A novel synthesis of high-loaded antimicrobial drug-silica co-assembled particles (DSPs) were recently developed through co-assembly of silica and octenidine dihydrochloride [91]. It has been reported to enhance the antimicrobial performance of EndoSequence BC sealer [61].

Engineered bioactive chitosan nanoparticles have been shown to effectively inactivate bacterial biofilm and disrupt its extracellular polymeric matrix [90]. They were reported to eliminate mono-species and multi-species biofilms [92] in a time-dependent interaction and present a potential antimicrobial/antibiofilm agent for root canal disinfection [93,94]. Carboxymethyl chitosan has been reported as a surface modifier of dentin matrix to enhance antibacterial efficacy [95]. Engineered chitosan-based nanoparticles, as a bioactive biopolymer, might alter the host inflammatory response of macrophages and promote healing [96]. Chitosan has been widely incorporated in biological scaffolds for bone regeneration [97,98] and tissue engineering [99,100]. DaSilva et al. [23] incorporated chitosan nanoparticles into the zinc oxide-eugenol sealer and the mixer inhibited biofilm formation within the sealer-dentin interface. Given their high biocompatibility and wide application in bioengineering, chitosan nanoparticles should have further potential to be used as a supplement to different endodontic bioceramics.

Other nanoparticles, including silver nanoparticles [101] and bismuth lipophilic nanoparticles [102] were also used in endodontics and showed antimicrobial activities against single-species biofilms. However, the contribution of their cytotoxicities to the bioceramic materials is concerning and requires further investigation.

## 6. Outlook

The application of bioceramic technology has optimized endodontic disinfection, providing a promising direction for the preservation of patients’ teeth. The introduction of different obturation techniques, from traditional cold lateral condensation, warm vertical condensation, to the recently popular single-cone technique, allows for the sealer to be the main component of the root filling. Efforts have been made to use core material as the delivery device to allow hydraulic movement of the bioceramic sealer into all the irregularities of the root canal system to achieve an ideal obturation. The biocompatibility, bioactivity, and antimicrobial, and antibiofilm properties have made bioceramics a promising material used in endodontics. However, persistent and recurrent apical periodontitis are still challenging enemies for current endodontic disinfection strategies. A higher level of evidence with randomized clinical trials is needed to prove the efficacy of bioceramic materials in the long term.

## Data Availability

No new data were created or analyzed in this article. Data sharing is not applicable to this article.
